# Synthesis of Cellulose-2,3-bis(3,5-dimethylphenylcarbamate) in an Ionic Liquid and Its Chiral Separation Efficiency as Stationary Phase

**DOI:** 10.3390/ijms15046161

**Published:** 2014-04-11

**Authors:** Runqiang Liu, Yijun Zhang, Lianyang Bai, Mingxian Huang, Jun Chen, Yuping Zhang

**Affiliations:** 1 College of Plant Protection, Hunan Agricultural University, Changsha 410128, China; E-Mail: lrq@hist.edu.cn; 2 College of Chemistry and Chemical Engineering, Henan Institute of Science and Technology, Xinxiang 453003, China; E-Mails: zhangyijun@hist.edu.cn (Y.Z.); huangmingxian@hist.edu.cn (M.H.); junchen713@hist.edu.cn (J.C.); 3 Hunan Academy of Agricultural Sciences, Changsha 410125, China

**Keywords:** 1-allyl-3-methyl-imidazolium chloride, synthesis, Cellulose-2,3-bis(3,5-dimethylphenylcarbamate), high performance liquid chromatography, chiral stationary phase, enantioseparation

## Abstract

A chiral selector of cellulose-2,3-bis(3,5-dimethylphenylcarbamate) (CBDMPC) was synthesized by reacting 3,5-dimethylphenyl isocyanate with microcrystalline cellulose dissolved in an ionic liquid of 1-allyl-3-methyl-imidazolium chloride (AMIMCl). The obtained chiral selector was effectively characterized by infrared spectroscopy, elemental analysis and ^1^H NMR. The selector was reacted with 3-aminopropylsilanized silica gel and the CBDMPC bonded chiral stationary phase (CSP) was obtained. Chromatographic evaluation of the prepared CSPs was conducted by high performance liquid chromatographic (HPLC) and baseline separation of three typical fungicides including hexaconazole, metalaxyl and myclobutanil was achieved using n-hexane/isopropanol as the mobile phase with a flow rate 1.0 mL/min. Experimental results also showed that AMIMCl could be recycled easily and reused in the preparation of CSPs as an effective reaction media.

## 1. Introduction

Cellulose derivatives provide broad recognitions for a wide range of enantiomers, which makes them one of the most important chiral stationary phases (CSPs) in the field of separation sciences. To develop new application, synthesis methods of cellulose derivatives have received considerable attention in the field of enantioseparation [1,2]. However, the poor solubility of cellulose in common organic solvents and water has held back its functionality effectively. Okamoto *et al.* have ever developed some typical CSPs including coated amylose- and cellulose-derived CSPs, bonded amylose- and cellulose-derived CSPs [3–7]. Zhang group also reported the preparation of two kinds of CSPs by coating and bonding cellulose-tris (3,5-dimethylphenylcarbamate) on the surfaces of aminopropyl-functionalized silica gel, respectively [8]. The tritylation of cellulose derivatives in the synthesis of these CSPs is generally carried out through the reaction between its hydroxyl groups and reactive isocyanates in pyridine. After the activated cellulose is transferred to dry pyridine, a slurry is then formed that becomes homogenous as the reaction progresses. Generally, pyridine used as the reaction solvents has certain disadvantages, such as instability, high toxicity, difficulty in recovery and high cost, to a certain degree. Therefore, the development of environment-friendly, low-cost reaction solvents for synthesizing homogeneous derivatives of cellulose is of great significance for the preparation of high quality cellulose derivative products. Ionic liquid is an organic salt which is liquid at room temperature. It has attracted wide attention in the field of chemistry for its non-volatility, low melting point, adjustable structure and properties, and other advantages [9]. Recently, Granström *et al.* synthesized selectively protected 2,6-di-*O*-(4-methoxytrityl)cellulose in an ionic liquid of 1-allyl-3-methyl-imidazolium chloride (AMIMCl). It showed that AMIMCl not only functions as solvents for cellulose, but also be capable of increasing the reactivity [10]. Xu *et al.* synthesized cellulose dehydroabietate by the *O*-acylation reaction of cellulose with dehydroabietic acid chloride using ionic liquid 1-butyl-3-methylimidazolium bromide as a solvent [11]. These findings open a new field for investigating and developing the cellulose solvent system.

In the present study, cellulose-2,3-bis(3,5-dimethylphenylcarbamate) (CBDMPC) was synthesized in ionic liquid AMIMCl and the CBDMPC bonded chiral stationary phase (CSP) was obtained and evaluated.

## 2. Results and Discussion

### 2.1. Synthesis and Characterization of CBDMPC

**Figure 1.**
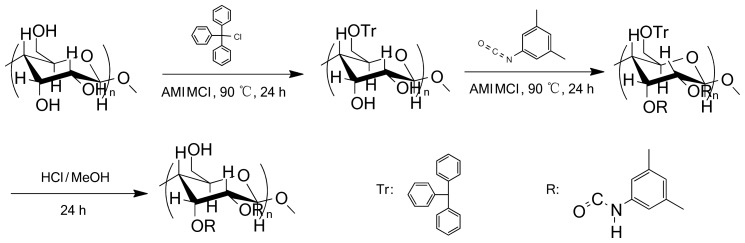
Synthesis of cellulose-2,3-bis(3,5-dimethylphenylcarbamate) (CBDMPC).

**Figure 2.**
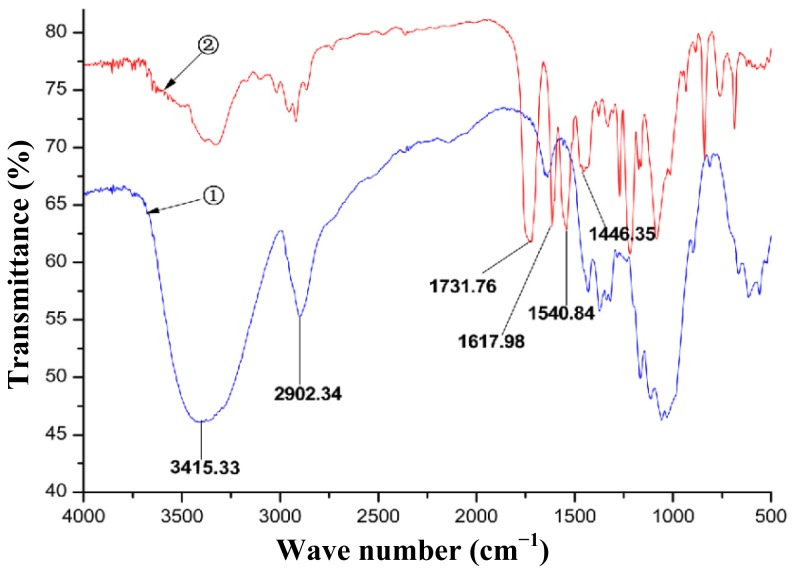
IR spectra of microcrystalline cellulose and CBDMPC. ➀ microcrystalline cellulose; ➁ CBDMPC.

The synthesis of CBDMPC includes tritylation, carbamation and the removal of methoxy-substituted trityl groups (Figure 1) [12]. In the present study, the microcrystalline cellulose was firstly dissolved in ionic liquid AMIMCl to form homogeneous solution; Secondly, the solution was treated with trityl chloride. Because of steric demands, trityl chloride reacts preferentially with the primary hydroxyl group at the *O*-6 position on the cellulose backbone rather than with either of the secondary hydroxyl groups at the *O*-2/3 positions [13]; Thirdly, the reaction solution was added 3,5-dimethylphenyl isocyanate to produce carbamate; Finally, the hydrolysis of the reaction solution in hydrochloride acid and methanol mixture afforded the titled compound. FTIR spectra of microcrystalline cellulose and CBDMPC were compared (Figure 2). A significant weaker -OH absorption peak appeared at 3415.33 cm^−1^ in the spectrum of CBDMPC. It indicated that most –OH groups of cellulose were replaced. The C–H stretching vibration peak of –CH_3_ occurred at 2902.34 cm^−1^, the absorption peaks of the benzene ring were observed at 1617.98, 1540.84 and 1446.35 cm^−1^, and the absorption peak of the carbonyl group occurred at 1731.76 cm^−1^. The results demonstrate that the –OH groups on microcrystalline cellulose had reacted with 3,5-dimethylphenyl isocyanate. Preparation of CSPs was carried out by bonding CBDMPC on the surfaces of 3-aminopropyltriethoxysilane -modified silica gel (for detail, refer to experiment Section 3.3).

The obtained CBDMPC was also determined by the ^1^H NMR (400 MHz, 50 °C, DMSO-*d*_6_) and the chemical shifts (δ, ppm) were assigned as follows: 8.6–9.0 (N–H), 6.3–7.0 (Ar–H), 3.6–5.0 (Glucose–H) and 2.0–2.1 (Ar–CH_3_). Moreover, according to the molecular formula (C_24_H_28_O_7_N_2_)_n_ of CBDMPC, the theoretical percentage of each element could be calculated as follows: C 63.18%, H 6.18% and N 6.14%. In contrast, the measured values for each element by elemental analysis were C 64.57%, H 6.26% and N 6.56%, respectively. Little difference between the theoretical and measured values for each element further demonstrated that cellulose-2,3-bis(3,5-dimethylphenylcarbamate) was successfully synthesized.

### 2.2. Enantioseparation of Three Fungicides Using the Prepared Chiral Column

**Figure 3.**
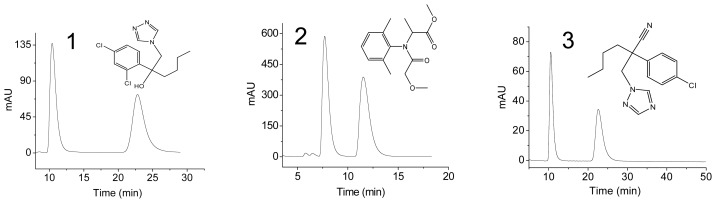
Optimal chromatograms of three fungicides and their molecular structures. Peak identification: **1** hexaconazole; **2** Metalaxyl; **3** Myclobutanil. Chromatographic conditions: Enantioseparation of hexaconazole and myclobutanil was conducted using *n*-hexane/isopropanol (70/30, *v*/*v*) as the mobile phases at a flow rate of 1.0 mL/min, whilst enantioseparation of metalaxyl was conducted using *n*-hexane/isopropanol (75/25, *v*/*v*) as the mobile phases at a flow rate of 1.0 mL/min.

In order to investigate the ability of chiral separation of CBDMPC-bonded CSPs, three typical fungicides including hexaconazole, metalaxyl and myclobutanil were selected for the evaluation in HPLC. Chromatographic conditions were selected with a flow rate of 1.0 mL/min and a column temperature of 25 °C. The UV detection wavelength was set at 230 nm for hexaconazole, 214 nm for metalaxyl, and 210 nm for myclobutanil, respectively. The dead time was determined through the injection of 1,3,5-tri-*tert*-butylbenzene. Some chromatographic parameters including the resolution (*R*_s_), capacity factor (*k*′), separation factor (*α*) for three chiral fungicides were listed in Table 1. It was easily understood that the values of *k*′, *α* and *R*_s_ decreased with the increase of isopropanol from 5%–30% in the mobile phases. This is because that hydrogen bond force of the sample molecules in the mobile phase increased with the decrease in isopropanol content, which decreased the sorption and desorption rates of the sample molecules in the mobile phase, thus increasing the separation power. The optimal chromatograms of three chiral fungicides are typically shown in Figure 3.

Compared with the coated CSPs, the bonded CSPs may enhance the chiral separations due to their more choices of mobile phases. Here, in order to examine the stability of the self-made chiral column, about 1%–5% (*v*/*v*) THF and CHCl_3_ as a polar modifier were added to the mobile phases (*n*-hexane/isopropanol), respectively. After long runs of chromatographic operation with a flow rate of 2.0 mL/min at 35 °C, no abnormal phenomena, such as reduced column efficiency for the selected fungicides and unstable baseline, was observed, demonstrating CBDMPC were bonded on the silica gel tightly.

### 2.3. Recycling and Characterization of the AMIMCl

In view of environmental conservation and economics of the process, the solvent ionic liquid should be recycled and reused. In our study, at the end of carbamation of cellulose, the reaction mixture was poured into methanol, the CDMPC was precipitated and washed with methanol, and the filtrate was simply evaporated, giving a clean sample of AMIMCl, which was confirmed by IR and MS Spectroscopy. The fresh ionic liquid and recovered ionic liquid have almost the same spectral shapes, demonstrating that the purity of recovered ionic liquid is as high as that of the fresh ionic liquid (for detail, refer to supplementary).

## 3. Experimental Section

### 3.1. Chemicals and Equipments

AMIMCl was purchased from Shanghai Cheng Jie Chemical Co., Ltd. (Shanghai, China). Spherical silica gel (Kromasil, 5 μm, pore size 10 nm) was purchased from Akzo Noble (Nacka, Sweden). Microcrystalline cellulose (Avicel, *DP* = 200), 3-aminopropyltriethoxysilane (KH-550), hexamethylene diisocyanate, and 3,5-dimethylphenyl isocyanate were purchased from Aladdin Chemistry Co., Ltd. (Shanghai, China). Hexaconazole standard sample (95%) and metalaxyl standard sample (98%) were provided by Hunan Institute for Food and Drug Control (Changsha, China). Myclobutanil standard sample (98%) was provided by Hunan Research Institute of Chemical Industry (Changsha, China). The *n*-hexane, isopropanol, tetrahydrofuran (THF) and trichloromethane used for mobile phase were HPLC grade, and purchased from Tianjin Chemical Reagent Co., Inc. (Tianjin, China). All mobile phases were filtrated through a 0.45 μm filter membrane and degassed by ultrasonication prior to use.

Chromatographic measurements were made on a Agilent 1100 HPLC system (Agilent Technologies, Inc., Walbronn, Germany) equipped with a quaternary pump, a vacuum degasser module, a Rheodyne 7725i injector with a 20 μL sample loop, a temperature controlled column compartment and a variable wavelength UV detector. Fourier transform infrared spectroscopy (FTIR) spectra were recorded in the range 400–4000 cm^−1^ with 4 cm^−1^ resolution with a BRUKER TENSOR27 system (Bruker Scientific Technology Co., Ltd., Karlsruhe, Germany). The 400 MHz ^1^H NMR spectra were measured by a BRUKER ARX 400 spectrometer (Bruker Scientific Technology Co., Ltd., Karlsruhe, Germany).

### 3.2. Synthesis of Cellulose-2,3-bis(3,5-dimethylphenylcarbamate)

One gram of dry microcrystalline cellulose (degree of polymerization of 200) was dissolved in 20 mL of ionic liquid AMIMCl at 90 °C, then 3.50 g of triphenylchloromethane was added into the solution. The solution was reacted at 90 °C for 24 h under vigorously stirring. 6.6 mL of 3,5-dimethylphenyl isocyanate was added into the solution, and reacted for another 48 h at 90 °C. After reaction was completed, the mixture was cooled and poured into 400 mL of anhydrous methanol containing 3.3 mL of concentrated hydrochloric acid, and the mixture was stirred at room temperature for 24 h. The white precipitate was separated by suction filtration and washed several times with large amounts of anhydrous methanol. The collected solid was dried under vacuum at 50 °C to obtain the CBDMPC with a yield of 85%.

### 3.3. Preparation of Bonded-Type Chiral Stationary Phase

In brief, 8 g of spherical silica gel was transferred into a 150 mL three-necked flask, then dried in vacuum at 110 °C for 24 h, cooled to room temperature, and added with 80 mL of anhydrous toluene and 16 mL of 3-aminopropyltriethoxysilane. The resulting mixture was allowed to react at reflux for 48 h. The entire process was conducted under a nitrogen atmosphere. The mixture was filtered after cooling, and then washed with an appropriate amount of toluene, methanol, diethyl ether, petroleum ether. The product was dried in vacuum at 60 °C for 5 h to obtain white 3-aminopropylsilanized silica gel [14].

Nought point eight three gram of CBDMPC was dissolved in 10 mL of THF, followed by the addition of 3.30 g 3-aminopropylsilanized silica gel. The solution was dried for 8 h under vacuum at 60 °C so as to remove the solvent. Afterwards, 30 μL of hexamethylene diisocyanate and 10 mL of anhydrous toluene were added and thoroughly mixed. The mixture was allowed to react at 100 °C for 24 h, and then added with 4.5 mL of 3,5-dimethylphenyl isocyanate. The resulted mixture was allowed to react at reflux for another 48 h and then filtered to obtain a solid product. Cellulose derivatives were then regioselectively bonded to silica gel on their glucose units [14]. The product was subjected to Soxhlet extraction for 24 h using THF and methanol, respectively, and then dried in vacuum at 60 °C for 24 h to obtain the final CSP products.

### 3.4. Packing

Using isopropanol as the slurry solvent and *n*-hexane/isopropanol (90/10, *v*/*v*) solution as the displacement fluid, the prepared CSP was packed into a stainless-steel column (250 mm × 4.6 mm) at a pressure of 7000 Psi.

## 4. Conclusions

In conclusion, the green solvent, ionic liquid AMIMCl can absolutely replace the traditional solvent pyridine in the synthesis of the chiral selector cellulose-2,3-bis(3,5-dimethylphenylcarbamate). Successful synthesis of the chiral selector was proved by three characterization methods: IR, ^1^H NMR and elemental analysis. Chromatographic evaluation performed on the self-made chiral column in HPLC demonstrated that our developed method is effective for the green synthesis of CSPs.

## Supplementary Information



## Figures and Tables

**Table 1.** Effect of isopropanol content on enantioseparation of three fungicides.

Compound	*n*-Hexane/Isopropanol (*v*/*v*)	*k*′_1_	*k*′_2_	*α*	*R*_s_
Hexaconazole	70/30	2.81	7.35	2.62	5.23
	75/25	3.68	9.70	2.64	5.50
	80/20	4.84	13.18	2.73	6.05
	85/15	7.52	21.14	2.81	9.30
	90/10	12.10	35.51	2.94	11.23

Metalaxyl	75/25	1.92	3.37	1.76	2.64
	80/20	2.58	4.73	1.83	3.62
	85/15	3.27	6.09	1.86	3.74
	90/10	4.84	9.45	1.95	3.89
	95/5	9.59	20.25	2.11	4.37
	98/2	22.02	49.12	2.23	11.74

Myclobutanil	70/30	2.85	7.28	2.55	5.01
	75/25	3.69	9.50	2.57	5.29
	80/20	5.29	13.97	2.64	6.08
	85/15	6.86	19.84	2.89	6.39
	90/10	12.01	34.89	2.91	7.09
