# Supplementary material for: Synthesis of Cellulose-2,3-bis(3,5-dimethylphenylcarbamate) in an Ionic Liquid and Its Chiral Separation Efficiency as Stationary Phase
Source: Int J Mol Sci. 2014 Apr 11;15(4):6161–8. doi: 10.3390/ijms15046161 (PMC4013621; doi:10.3390/ijms15046161)
Supplement: Supplementary file 1 [file ijms-15-06161-s001.pdf]

Supplementary Information

Recycling and Characterization of the AmimCl

In view of environmental conservation and economics of the process, the solvent ionic liquid should be recycled and reused. In our study, at the end of esterification of cellulose, the reaction mixture was poured into methanol, the CDMPC was precipitated and washed with methanol, and the filtrate was simple evaporated, giving a clean sample of AmimCl, the percent of recovery was about 94%, which was confirmed by IR (Figure S1) and MS Spectroscopy (Figure S2). The fresh ionic liquid and recovered ionic liquid have almost the same spectral shapes, demonstrating that the purity of recovered ionic liquid is as high as that of the fresh ionic liquid. For example, 3056 and 1423 cm^{-1} correspond to stretch bending vibration peak on allylic C=CH2 and C=C, respectively, 1165, 997 and 943 cm^{-1} of C–H bending vibration peaks in the imidazole ring correspond to rocking vibration peak in C–H plane of the allylic –CH=CH2 and rocking vibration peak in C–H plane of allylic –CH=CH2, respectively. In addition, there always exists 3360 cm^{-1} peak of water impurity because AmimCl has a strong absorption of moisture, 1562 and 1165 cm^{-1} are characteristic of imidazolium cations peaks, as is to be observed in many imidazolium ionic liquids. As shown in Figure 5, positive ion FAB mass spectrometry tests were conducted in fresh ionic liquids and recovered ionic liquids respectively, giving the molecular ion peaks of the abundance of 100 both appeared at 123 locations, as is consistent with $[Amim]^+$ molecular ion mass. Under the same reaction,when the recycled AmimCl was used as solvent in Synthesis of cellulose-2,3-bis(3,5-dimethylphenylcarbamate), the CBDMPC also with a yield of about 85%.

Figure S1. IR spectrums of ionic liquids before and after reaction. (A) Fresh ionic liquid; (B) Recovered ionic liquid.

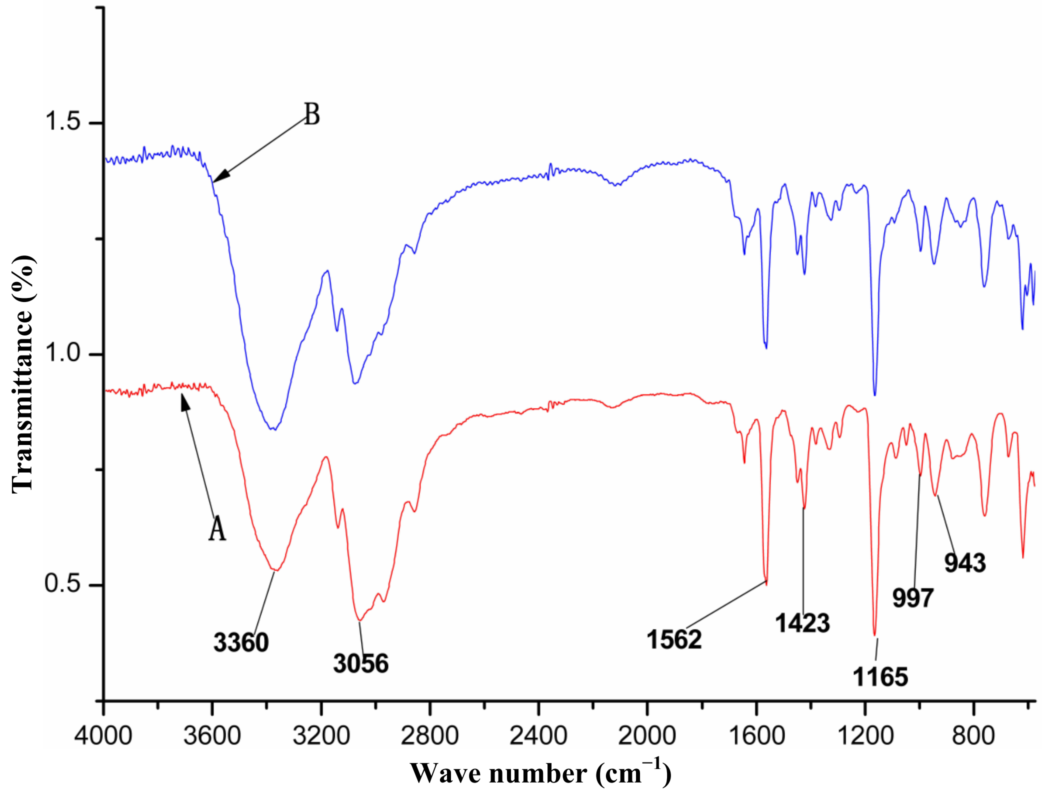

Figure S2. Mass spectrums of ionic liquids before (**A**) and after (**B**) reaction.

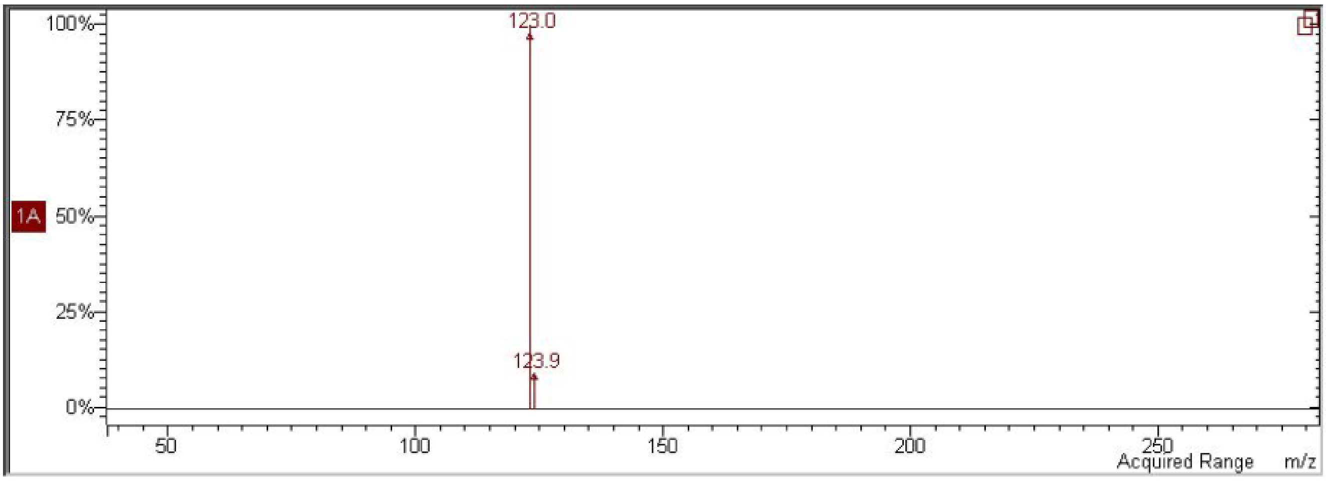

(**A**)

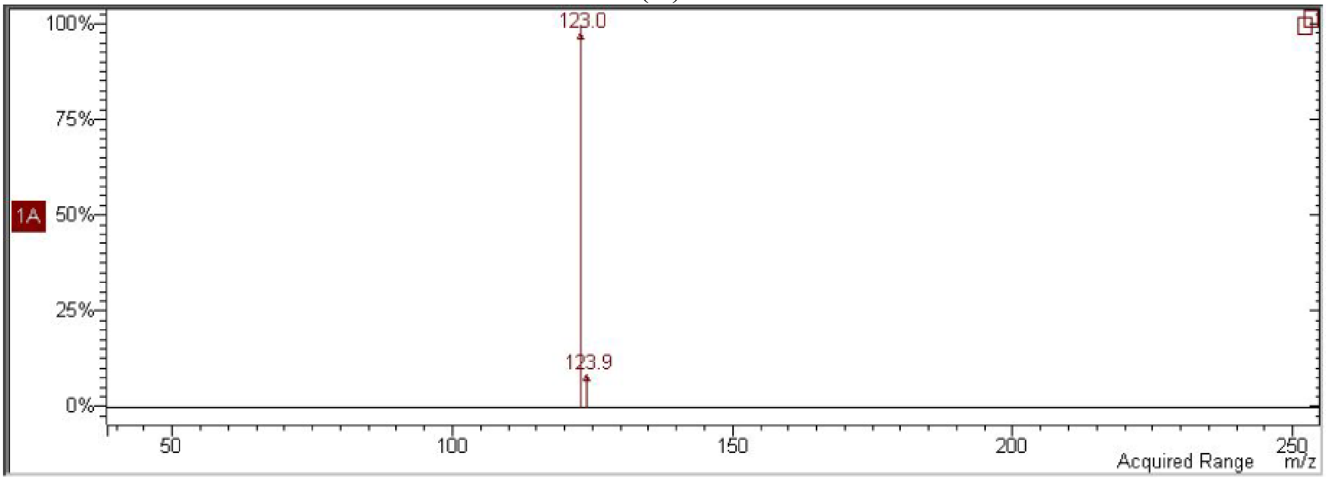

(**B**)

© 2014 by the authors; licensee MDPI, Basel, Switzerland. This article is an open access article distributed under the terms and conditions of the Creative Commons Attribution license (http://creativecommons.org/licenses/by/3.0/).
